# Impact of oral anticoagulation therapy on postoperative atrial fibrillation outcomes: a systematic review and meta-analysis

**DOI:** 10.1186/s12959-021-00342-2

**Published:** 2021-11-19

**Authors:** Mariana Fragão-Marques, Francisco Teixeira, Jennifer Mancio, Nair Seixas, João Rocha-Neves, Inês Falcão-Pires, Adelino Leite-Moreira

**Affiliations:** 1 Cardiovascular Research and Development Center, Faculty of Medicine of the University of Porto, 4200 Porto, Portugal; 2 São João University Hospital Center, Porto, Portugal; 3 Faculty of Medicine of the University of Porto, Porto, Portugal; 4 Vila Nova de Gaia Hospital Center, Vila Nova de Gaia, Portugal

**Keywords:** Atrial fibrillation, Cardiac surgery, Anticoagulation, Long-term outcome

## Abstract

**Background:**

Post-operative atrial fibrillation (POAF) is the most common complication after cardiac surgery. Recent studies had shown this phenomenon is no longer considered transitory and is associated with higher risk of thromboembolic events or death. The aim of this study was to systematically review and analyze previous studies comparing oral anticoagulation therapy with no anticoagulation, regarding these long-term outcomes.

**Methods:**

PubMed/MEDLINE, EMBASE, Web of Science and Cochrane Database were systematically searched to identify the studies comparing the risk of stroke, or thromboembolic events or mortality of POAF patients who received anticoagulation compared with those who were not anticoagulated. Incidence of stroke, thromboembolic events and all-cause mortality were evaluated up to 10 years after surgery. Time-to-event outcomes were collected through hazard ratio (HR) along with their variance and the early endpoints using frequencies or odds ratio (OR). Random effect models were used to compute statistical combined measures and 95% confidence intervals (CI). Heterogeneity was evaluated through Q statistic-related measures of variance (Tau^2^, I^2^, Chi-squared test).

**Results:**

Eight observational cohort studies were selected, including 15,335 patients (3492 on Oral Anticoagulants (OAC) vs 11,429 without OAC) that met the inclusion criteria for qualitative synthesis. Patients had a wide gender distribution (38.6–82.3%), each study with a mean age above 65 years (67.5–85). Vitamin K antagonists were commonly prescribed anticoagulants (74.3–100%). OAC was associated with a protective impact on all-cause mortality at a mean of 5.0 years of follow-up (HR is 0.85 [0.72–1.01]; *p* = 0.07; I^2^ = 48%). Thromboembolic events did not differ between the two treatment arms (HR 0.68 [0.40–1.15], *p* = 0.15).

**Conclusion:**

Current literature suggests a possibly protective impact of OAC therapy for all-cause mortality in patients with new-onset atrial fibrillation after cardiac surgery. However, it does not appear to impact thromboembolism rate.

**Supplementary Information:**

The online version contains supplementary material available at 10.1186/s12959-021-00342-2.

## Introduction

Postoperative atrial fibrillation (POAF) is the most common complication after cardiac surgery, with a global incidence of 20–40%. Although its pathophysiologic mechanism is still uncertain, some progress has been made. Cardiac surgery is a stressful event which generates a chain of inflammatory reactions [1], with pro-inflammatory cytokines and increased oxidative stress. Such inflammation affects atrial conduction during atrial fibrillation by changing sodium channel function through the reduction of sodium currents and consequent upstroke velocity [2–4].

Moreover, one third of patients after coronary artery bypass grafting (CABG) and almost half of patients after valve repair/replacement (VR) develop atrial fibrillation after a cardiac procedure, usually occurring early in the recovery period [5, 6]. Although this arrhythmia was previously thought as transitory and benign, patients who develop the arrhythmia have longer intensive care unit hospitalizations, higher healthcare costs, and an increased risk of postoperative complications, namely stroke, thromboembolic events and mortality, both intrahospital and at 6 months [7–9].

While the relationship between short-term outcomes and POAF is well defined, only a few studies have demonstrated that POAF is associated with higher long-term stroke risk and mortality, contrasting to what was previously thought of POAF as a transitory and nonthreatening event [3, 10]. Indeed, surgical and anaesthetic techniques have improved over time, although patients are older and have a higher prevalence of comorbidities, possibly implying an increased structural left atrium remodelling [11–13]. Furthermore, evidence on anticoagulation therapy in this subgroup of patients is scarce, although some studies have analyzed the relationship of anticoagulation therapy in POAF management with the occurrence of adverse outcomes, namely thromboembolism and all-cause mortality [14–18]. Because of the older age of cardiac surgery patients, risks and benefits of OAC (Oral Anticoagulant) therapy should be carefully considered.

European guidelines for the management of atrial fibrillation are very clear and encourage the use of anticoagulation in AF patients for stroke prophylaxis [19]. Conversely, management of POAF is still a topic of debate in the scientific community. On one hand, 2014 AHA/ACA guidelines recommend the use of beta-blockers and nondihydropyridine calcium channel blockers, while ESC 2020 guidelines suggest amiodarone or vernakalant have been efficient in converting postoperative AF to sinus rhythm. On the other hand, the use of anticoagulation lacks good quality evidence, with no randomized clinical trials available and few observational studies. Thus, European guidelines leave the decision of anticoagulation therapy to the physician, while American guidelines (AHA/ACA) do not mention any course of action on this matter [20, 21].

The aim of this systematic review/meta-analysis was to investigate if the use of anticoagulation in patients who developed POAF undergoing cardiac surgery has an association with lower rates of long-term thromboembolic events, stroke, major bleeding and all-cause mortality; and to identify subgroups of POAF patients that can benefit more from OAC therapy.

## Methods

The study protocol was registered in PROSPERO (PROSPERO ID: CRD42020208229) in accordance with standard reporting conventions. PRISMA guidelines were used in the writing of this manuscript.

### Data sources and search strategy

A MEDLINE, EMBASE, Web of Science and Cochrane Database search was performed to find evidence up to the 5th August 2021 on stroke prevention with oral anticoagulants in patients who developed atrial fibrillation after cardiac surgery. The search query was constructed using the keywords *“POAF”, “new onset postoperative atrial fibrillation”, “new onset atrial fibrillation”, “postoperative atrial fibrillation”, “anticoagulants” and “stroke”.* The detailed search query is reported in supplementary materials (Table 1S).

### Study selection

One reviewer independently screened search records for inclusion (FT) and another checked decisions (MFM). Titles and abstracts were screened at this stage and relevant studies were selected for full-text analysis. Inclusion criteria included randomized clinical trials and cohort studies on patients submitted to cardiac surgery who developed postoperative atrial fibrillation and received oral anticoagulation therapy versus no anticoagulation. Selected studies had to include stroke, a combined outcome including stroke (example: major adverse cardiovascular events (MACE), thromboembolic events), or all-cause mortality. Exclusion criteria included manuscripts in a language other than English, no full text available, duplicate manuscripts, and absence of outcomes of interest in the published manuscript.

### Data extraction and quality assessment

Two reviewers (MFM, FT) blindly and independently checked the full texts, decided on the inclusion of individual studies and extracted data about study characteristics and event rates. Disagreements between reviewers were decided by the most senior reviewer. Extracted data included event rates of long-term 1) thromboembolism, a composite outcome of ischemic stroke, transient cerebral ischemia, and thrombosis or embolism in peripheral arteries; 2) stroke; 3) all-cause mortality; and 4) major bleeding. Estimates were presented as adjusted Hazard Ratios (HR) and respective 95% Confidence Intervals (CI).

Risk of bias was assessed by two blinded reviewers (MFM, FT), independently, using the Cochrane Risk Of Bias in Non-randomized Studies - of Interventions (ROBINS-I) tool (all selected studies were observational) [22]. Evaluated bias domains included bias due to confounding, selection bias, bias in classification of interventions, bias due to deviations from intended interventions, bias due to missing data, bias in measurement of outcomes and bias in selection of the reported result.

### Data analysis

Assessment of reporting bias was performed with the Egger’s regression test and respective funnel plot. Heterogeneity was evaluated through Q statistic-related measures of variance (Tau^2^, I^2^, Chi-squared test), and a random-effects model with an inverse variance method was preferred to compute estimates for the summary effect. Forest plots were used to display results from the meta-analysis, where the measure of effect for each study is represented by a square and the respective areas are proportional to study weight.

A *p* value inferior to 0.05 was considered statistically significant for all analyses. Computations were conducted using Review Manager 5.3 and Stata 16.1.

## Results

**Fig. 1:**
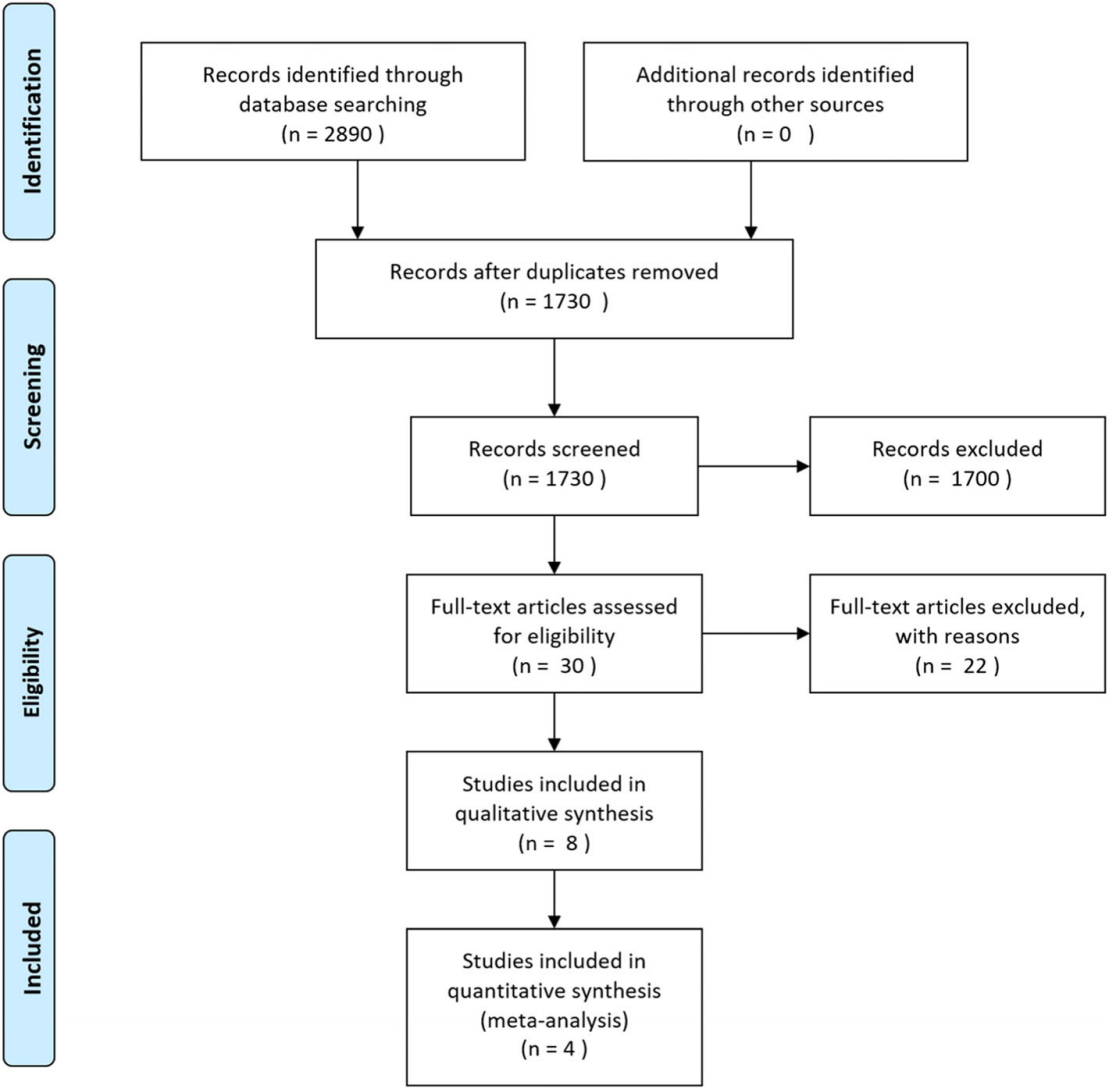
Flowchart of the screening and selection process

A total of 2890 records were identified through database searching, of which 1730 were kept after duplicates were removed. Thirty publications were assessed for eligibility through their full texts, with 8 manuscripts selected for qualitative synthesis. Two publications were excluded from quantitative analysis, since the number of patients and events was extremely low, with two additional studies excluded because their patient population was submitted to transcutaneous procedures (see below). Fig. 1 depicts the study selection process.

Included research papers were published between 2010 and 2021, with a wide range of locations (Denmark, USA, South Korea, Poland, United Kingdom, Australia, Brazil, Italy, India). All papers were observational and based on a prospective cohort (Table 1). The total number of included patients with POAF for quantitative synthesis was 12,733 (2978 with Oral Anticoagulants (OAC) vs 9755 without OAC). Vitamin K antagonists represented the majority of prescribed oral anticoagulants across all studies (74.3–100%), although Yoon et al. 2019 lacked information concerning this issue [27].

**Table 1:** Included studies in qualitative synthesis

**First Author**	**Year**	**Country**	**Patient Selection**	**Study Design**	**Anticoagulation**	**POAF definition**				**POAF (N)**	**OAC vs No Anticoagulants**	**Number**	**Age**	**Sex % (Males)**	**Type of Surgery**		
															**CABG**	**Valve**	**CABG + Valve**
**Butt et al.** **[**23]	2018	Denmark	1/1/2000–30/6/2015	Prospective Cohort	83.4% Warfarin	AF rhythm requiring either medical therapy or cardioversion during hospitalization in patients without previous history of AF episodes				2108	Vitamin K or non-vitamin K antagonists	175	Median (IQR): 69.2 (63.7–74.7)	82.3	X		
											No anticoagulant	1527					
**Vora et al.** **[**24**]**	2018	USA	01/11/2011–30/09/2015	Prospective Cohort	83.0% Warfarin	New-onset AF following TAVR				1138	Oral Anticoagulant	329	Median (IQR): 85 (78–88)	38.6		TAVR	
					15.2% Factor Xa inhibitor												
					1.8% Dabigatran						No anticoagulant	809					
**Butt et al.** **[**25**]**	2019	Denmark	01/01/2000–30/06/2015	Prospective Cohort	97.6% Warfarin	AF rhythm requiring either medical therapy or cardioversion during hospitalization in patients without previous history of AF				675	Oral Anticoagulant	420	Median (IQR): 71 (65–77)	59.3		Aortic and/or Mitral Valve Repair/ Replacement	
											No anticoagulant	255					
**El-Chami et al.** **[**26**]**	2010	USA	1/1/1996–31/12/2007	Retrospective analysis of a Prospective Cohort	100% Warfarin	Occurence of new-onset POAF or atrial flutter requiring treatment				2985	Warfarin	613	Mean ± sd: 67.5 ± 9.5	73	X		
											No anticoagulant	2375					
**Yoon et al.** **[**27**]**	2019	South Korea	1/3/2010–1/2/2017	Prospective Cohort	Warfarin or Novel Oral Anticoagulant	Occurence of any episode of new-onset AF or flutter through hospitalization that lasted at least 30 s				31	Anticoagulant	11	Mean ± sd: 79.4 ± 5.0	52		TAVR	
											No anticoagulant	20					
**Taha et al.** **[**28**]**	2020	Sweden	1/1/2007–31/12/2015	Prospective Cohort	86.2% Vitamin K antagonists	POAF was defined as any new-onset atrial fibrillation during the first 30 postoperative days				7368	OAC	1770	Mean ± sd: 70 ± 8.0	81.5	X		
											No Anticoagulants	5598					
**Benedetto et al.** **[**29**]**	2020	Poland, UK, Austria, Australia, Brazil, Italy, India	06/2004–12/2007	Post hoc analysis of a Randomized Controlled Multicenter Trial	100% Warfarin	Occurence of any episode of AF or flutter (collectively called pAF for this analysis) after the index procedure through the time of discharge that lasted at least 30 s and was captured on a standard 12-lead ECG or cardiac telemetry				734	Warfarin	61	Mean ± sd: 66.41 ± 8.16	86,5	X		
											No Anticoagulant	662					
**Madsen et al.** **[**30**]**	2021	Denmark	1999–2016	Retrospective cohort	74.3% Vitamin K antagonists	AF within 30 days following STEMI				296	OAC therapy	113	Median (IQR): 71 (64–79)	69.9	PPCI		
											No Anticoagulant	183					
**First Author**	**Follow-up time**		**Thromboembolism**		**Stroke**	**All-cause Mortality**	**CHA2DS2-VASc**			**HAS-BLED**		**Major bleeding**			**Echocardiography data**		
										**OAC**	**No OAC**				**Left ventricle ejection fraction ≤ 30% n(%)**		
**Butt et al.** **[**23**]**	Median (IQR) 5.1 (2.2–9.2) years		HRa: 0.55 [0.32–0.95]		–	HRa: 1.09 [0.82–1.43]	**POAF:** mean ± sd: 3.2 ± 1.4			**POAF:** mean ± sd: 2.2 ± 1.1		–			**–**		
**Vora et al.** **[**24**]**	1 year		–		HRa: 1.12 [0.67–1.89]	HRa: 2.08 [1.56–2.78]	**POAF:** median (IQR): 5 [5, 6]			–		HRa: 0.77 [0.61–0.98]			46 (4.0)		
**Butt et al.** **[**25**]**	Median (IQR) 4.2 (2.0–7.1) years		HRa: 0.45 [0.22–0.90]		–	HRa: 0.64 [0.41–0.99]	**POAF:** mean ± sd: 2.9 ± 1.7			Mean ± sd: 2.1 ± 1.2	Mean ± sd: 2.2 ± 1.2	–			–		
**El-Chami et al.** **[**26**]**	Mean 6 years (range 0–12)		–		–	HRa: 0.78 [0.66–0.92]	–			–		–			–		
**Yoon et al.** **[**27**]**	12 months		OR: 0.67		–	–	**POAF:** mean ± sd: 4.1 ± 1.4			–		–			1 (3.2)		
**First Author**	**Follow-up time**		**Thromboembolism**		**Stroke**	**All-cause Mortality**	**CHA2DS2-VASc**			**HAS-BLED**		**Major bleeding**			**Echocardiography data**		
								**OAC**	**No OAC**	**OAC**	**No OAC**				**Left ventricle ejection fraction ≤ 30% n(%)**		
**Taha et al.** **[**28**]**	4.5 years (range 0–9)		HRa: 1.01 [0.77–1.33]		HRa: 1.08 [0.80–1.45]	HRa: [0.73–1.09]	**≥2**	n(%) 1637 (95.5)	n(%) 5201 (94.2)	–	–	HRa: 1.40 [1.08–1.82]			–		
							**≥4**	n(%) 925 (54.0)	n(%) 2738 (50.3)								
**Benedetto et al.** **[**29**]**	10 years		–		Cumulative incidence (OAC): 3.6% [95% CI 0.0–8.4%]	**–**	**POAF:** mean ± sd: 3.46 ± 1.31			–	–	Cumulative Incidence (OAC): 3.4 [95% CI 0.0–8.1]			21 (2.9)		
					Cumulative incidence (No OAC): 5.3% [95% CI 3.5–7.0%]							Cumulative Incidence (No OAC): 4.1 [95% CI 2.6–5.7]					
**Madsen et al.** **[**30**]**	Median (IQR) 4.7 (2.8–7.4) years		–		HRa: 0.70 [0.33–1.49]	HRa: 0.69 [0.47–1.00]	**≥2**	n(%) 105 (92.9%)	n(%) 169 (92.4%)	**≥3**: n(%) 77 (68.1)	**≥3:** n(%) 92 (50.3)	HRa: 1.31 [0.75–2.27]			–		

*HRa* adjusted Hazard Ratio with 95% Confidence Intervals

While POAF definitions included only new-onset episodes, Vora et al. 2018 and Yoon et al. 2019 considered all cases of POAF, in contrast with the remaining studies, which exclusively selected episodes requiring treatment. All studies had a mean age above 65 years (67.5–85), even though gender distribution varied significantly across studies (38.6–82.3% of males), with male gender being more common in CABG surgery. Selected research papers included both CABG [23, 26] and valve repair/replacement [24, 25, 27].

**Fig. 2:**
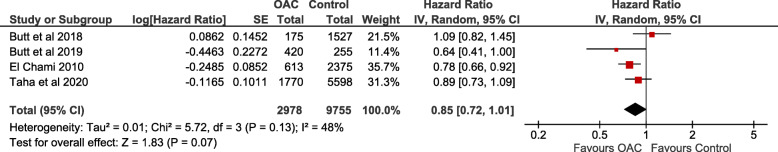
Pooled estimate of all-cause mortality with OAC therapy

Risk of bias is represented in Fig. 1S and Fig. 2S (supplementary content). Yoon et al. 2019 presented their results as a percentage of events between therapeutic groups, without an adjusted measure of effect or an adequate time-dependent analysis. In the OAC group, there was no description of which specific anticoagulants were used and only 2 thromboembolic events were reported. Thus, this study was considered as having a serious risk of bias and was excluded from quantitative analysis. Benedetto et al. 2020 is a post-hoc analysis of a well-designed randomized clinical trial, although it did not present time-dependent adjusted estimates; thus, this study was not included in the quantitative synthesis and had a serious risk of bias in domain 1, thus presenting an overall serious risk of bias. The remaining studies had a moderate risk of bias due to confounding, as all measures of effect were adjusted (see below), despite not being randomized controlled trials. The selected papers for quantitative synthesis all had time-dependent adjusted estimates and were exclusively from cohorts submitted to conventional cardiac surgery (Vora et al. and Madsen et al. were excluded). Moreover, considering papers with more than one reported outcome, Butt et al. 2018 and Butt et al. 2019 had into account competing risks in their outcome analysis. Taha et al. 2020, on the other hand, does not clarify this potential source of confounding.

Furthermore, there was little information concerning deviations from intended interventions or missing data. However, Butt et al. 2019 reported a decrease to 55.0% at 3 months of patients who were initially in the OAC treatment arm, to 31.7% at 6 months and 22.1% at 1 year of follow-up, being attributed a serious risk of bias concerning deviations from intended treatment. Benedetto et al. 2020 and El Chami et al. 2010 used multiple imputation to eliminate missing values, the latter considering absent data as missing at random; thus, a low risk of bias was considered in these cases. Apart from Yoon et al. 2019, all selected studies were classified as having an overall moderate risk of bias.

### All-cause mortality

All-cause mortality was reported in all selected studies for quantitative analysis. The median or mean follow-up varied between 4.2–6 years and the pooled HR is 0.85 [0.72–1.01] (*p* = 0.07; I^2^ = 48%), with 2978 vs 9755 (OAC vs control) analyzed patients (Fig. 2). Covariates used in the adjusted model are presented in Table 2S.

#### Publication bias

**Fig. 3:**
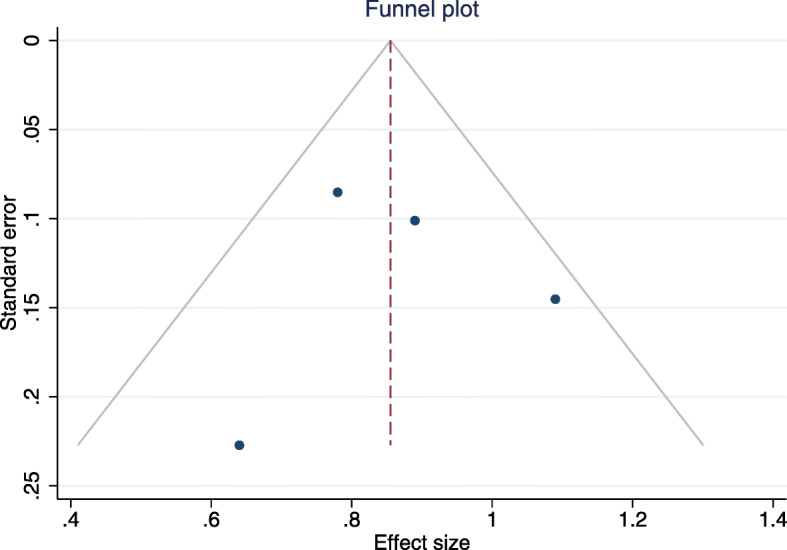
Funnel plot and Egger’s regression test

Publication bias for this outcome was evaluated by a funnel plot and Egger’s regression test (*p* = 0.85), which suggests absence of bias – Fig. 3.

### Thromboembolism

**Fig. 4:**
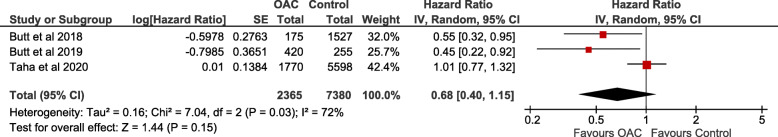
Pooled estimate of thromboembolism event with OAC therapy

Thromboembolism was reported in 3 of the selected studies [23, 25, 28] and was defined as a composite of ischemic stroke, transient cerebral ischemia, and thrombosis or embolism in peripheral arteries in all the research papers (Table 3S). A total of 2365 vs 7380 (OAC vs control) patients were included in the analysis and median follow-up time varied between 4.2–5.1 years. HR and 95% CI were adjusted for comorbidities and other concomitant medical therapy (Table 2S). The pooled estimate was HR 0.68 [0.40–1.15] (*p* = 0.15; I^2^ = 72%) and the respective forest plot is represented in Fig. 4.

### Stroke

Stroke was reported as a separate outcome in 3 studies – Madsen et al. (Primary Percutaneous Coronary Intervention - PPCI), Taha et al. (CABG) and Vora et al. (Transcatheter Aortic Valve Replacement - TAVR). The results were similar between studies, with no reported impact of OAC on stroke rates (Madsen et al. HR 0.70 [0.33–1.48]; Taha et al. HR 1.08 [0.80–1.46]; and Vora et al. HR 1.12 [0.67–1.87]). Table 2S presents the covariates included in each study’s multivariate cox regression.

### Major bleeding

This outcome was reported in 3 of the included studies (Madsen et al., Taha et al. and Vora et al.), with conflicting results between publications. Taha et al. found an association between OAC therapy and major bleeding (HR 1.40 [1.08–1.81]), with Madsen et al. reporting no significant impact of OACs in bleeding events (HR 1.31 [0.75–2.29]). However, Vora et al. reported a protective effect of this therapy – HR 0.77 [0.61–0.97]. The covariates used in each of the 3 studies are present in Table 2S and the respective definitions for major bleeding are in Table 3S.

## Discussion

In this meta-analysis, POAF patients treated with OACs appear to have a reduction in long-term all-cause mortality (*p* = 0.07, non-significant). Furthermore, these patients had a tendency towards a reduction in risk of long-term thromboembolic events (*p* = 0.15).

This work combined the available research papers on OAC therapy, resulting in estimates with a high number of patients (12,733 in total) corresponding to high-quality observational studies. AF has long been implicated in the incidence of stroke, with a fivefold increase in risk of ischemic stroke, namely, cardioembolic, which has particularly high mortality and residual disability [31]. Cryptogenic strokes may have AF as an underdiagnosed underlying cause, as demonstrated by monitoring with an insertable cardiac monitor in a randomized controlled trial [32]. In addition to stroke, prevention of systemic thromboembolic events is part of the fundamental management of AF [33]; AF represents a significant cause of mortality, with increasing incidence due to aging demographics [4, 34]. As far as AF management is concerned, anticoagulation therapy with vitamin K antagonists (VKA) reduces stroke and mortality by 64 and 26%, respectively [35]. On the other hand, the novel oral anticoagulants (NOAC) are a reasonable alternative for thromboembolic event prevention in non-valvular AF, with a 19% reduction in risk of stroke/systemic embolism, and 51% reduction in haemorrhagic stroke when compared with VKAs [36].

Nevertheless, paroxysmal AF classification remains elusive, with diverse patterns possibly requiring and presenting different treatments and outcomes [7, 37]. POAF is a subtype of paroxysmal AF linked to increased early stroke and 30-day mortality risk [38]. Concerning long-term results, POAF is associated with an increased risk of stroke and mortality, as demonstrated in a meta-analysis of patients submitted to both cardiac and noncardiac surgery, where the latter presented a higher risk of stroke when compared to cardiac surgery. These patients presented a 37% increase in risk of both long-term stroke and mortality [39]. In this work, OAC therapy appears to prevent long-term mortality (*p* = 0.07, non-significant), with a tendency to reduce long-term thromboembolic events (*p* = 0.15).

It is necessary to further understand if short and long POAF episodes present the same stroke risk, and if they benefit in a similar way from OAC therapy. Similarly, there is no evidence in which type of anticoagulation therapy should be used, as all research papers considered OAC therapy without discriminating NOAC vs VKA. Currently, guidelines recommend considering the overall presence of stroke and bleeding risk factors, although the definition of AF burden at which to initiate OAC therapy is poorly defined, and consequently, this knowledge gap results in significant variation in clinical practice [34, 40]. In this meta-analysis, bleeding risk varied between heterogeneous studies, which could be related both to the underlying disease (coronary artery disease or valvular disease) and to the type of procedure (CABG, PPCI or TAVR).

Thromboembolic events are one of the most serious complications of AF, and without anticoagulation therapy, stroke occurrence varies between 1.9 and 18.2%, depending on comorbidities [41]. The potential decrease in mortality in OAC-treated POAF patients could be related to cardiovascular events. Additionally, POAF is a risk factor for AF occurrence at follow-up, which could also help to explain these findings [41]. However, thromboembolism risk was not decreased significantly with OAC-therapy. Main causes of mortality in AF could be not only thromboembolic events but also heart failure (either ischemic or valvular) and bleeding due to anticoagulation therapy. The overall measure of effect (hazard ratio) in all-cause mortality was 0.85 [0.72–1.01] vs 0.68 [0.40–1.15] in thromboembolism. There were less studies reporting thromboembolism, with the ones reporting it, presenting estimates with greater standard errors, resulting in a larger confidence interval for thromboembolism when compared with all-cause mortality - [0.40–1.15] vs [0.72–1.01]. In addition, there could be a degree of under reporting of thromboembolic events when compared to mortality. Acute myocardial infarction is also a possible cause of death in these patients which was not included in the thromboembolism outcome (composite of ischemic stroke, transient cerebral ischemia, and thrombosis or embolism in peripheral arteries) [42]. These factors could have contributed to the discrepancy in results concerning all-cause mortality and thromboembolic events.

This systematic review and meta-analysis focused on different types of cardiac surgery with a relatively low number of studies, therefore increasing heterogeneity, despite including a high number of patients. Two of the manuscripts were from the same database, even though included patients were submitted to different types of surgery, limiting generalizability, counterbalanced by the wide range of locations (Denmark, USA, South Korea, Sweden, Poland, UK, India, Austria, Australia, Brazil, Italy). As all selected papers were observational, there is a risk of indication bias. Still, pooled estimates were adjusted for a vast number of covariates. Deviation from intended interventions was reported in three studies, with only one reporting a significant deviation in the treatment arm over time, which was accounted for in the statistical analysis [25]. Furthermore, Taha et al. did not have competing risks into account in their analysis, which could be a source of bias. Finally, although the estimates were adjusted, each study had a different pool of covariates in their regression models, increasing heterogeneity.

## Conclusions

Current literature suggests a possibly protective impact of OAC therapy for all-cause mortality in patients with new-onset atrial fibrillation after cardiac surgery. However, it does not appear to impact stroke, thromboembolism and major bleeding.

## Supplementary Information

**Additional file 1.** Supplementary Methods and Results.

## Data Availability

All data generated and analyzed are included in this research article.

## Abbreviations

AF: Atrial Fibrillation
AHA/ACA: American Heart Association/American College of Cardiology
POAF: Post-Operative Atrial Fibrillation
CABG: Coronary artery bypass grafting
CI: Confidence Intervals
ESC: European Society of Cardiology
FT: Francisco Teixeira
MACE: Major Adverse Cardiovascular Events
MFM: Mariana Fragão-Marques
NOAC: Novel Oral Anticoagulants
OAC: Oral Anticoagulants
OR: Odds Ratio
PPCI: Percutaneous Coronary Intervention
TAVR: Transcatheter Aortic Valve Replacement
VKA: Vitamin K Antagonists
VR: Valve repair/replacement
