# Supplementary material for: Impact of oral anticoagulation therapy on postoperative atrial fibrillation outcomes: a systematic review and meta-analysis
Source: Thromb J. 2021 Nov 19;19:89. doi: 10.1186/s12959-021-00342-2 (PMC8603521; doi:10.1186/s12959-021-00342-2)
Supplement: Supplementary file 1 — Additional file 1. Supplementary Methods and Results. [file 12959_2021_342_MOESM1_ESM.docx]

**Supplementary Content**

| Cochrane  Database | (POAF OR "new onset postoperative atrial fibrillation" OR "new onset atrial fibrillation" OR "postoperative atrial fibrillation") AND (anticoagulants OR stroke) |
| --- | --- |
| EMBASE | (POAF OR "new onset postoperative atrial fibrillation" OR "new onset atrial fibrillation" OR "postoperative atrial fibrillation") AND (anticoagulants OR stroke) |
| Web of Science | (POAF OR "new onset postoperative atrial fibrillation" OR "new onset atrial fibrillation" OR "postoperative atrial fibrillation") AND (anticoagulants OR stroke) |
| Pubmed | (POAF OR "new onset postoperative atrial fibrillation" OR "new onset atrial fibrillation" OR "postoperative atrial fibrillation") AND (anticoagulants OR stroke) |

**Table 1S.** Database search strategy.

**Table 1S.** Cochrane, EMBASE, Web of Science and Pubmed database search strategy.

**Table 2S.** Covariates adjusted in the multivariate cox regression models.

| Butt et al 2018 and 2019 | Ischemic heart disease  Heart failure  Thromboembolism  Peripheral vascular disease  Hypertension, coagulopathy  Bleeding  Diabetes  Malignancy  Chronic renal disease  Chronic obstructive pulmonary disease  Liver disease  Alcohol abuse  Statins  Aspirin  Adenosine diphosphate-receptor inhibitors  Dipyridamole  Nonsteroidal anti-inflammatory drugs |
| --- | --- |
| El Chami 2010 | Age  Sex  Race  Left ventricular ejection fraction  History of myocardial infarction  Index myocardial infarction  Congestive heart failure (chf)  New york heart association functional class  Stroke  Hypertension  Diabetes mellitus  Renal failure  Dyslipidemia  Smoking  Chronic obstructive lung disease  Peripheral vascular disease  Presence of left main disease (50%)  Last creatinine level  Dialysis status  Elective versus nonelective cabg,  Redo surgery  Presence of any valvular disorder or post-operative complications (myocardial infarction, stroke, intra-aortic balloon pump, Post-operative respiratory distress syndrome) |

**Table 2S.** (Continued)

| Vora et al 2018 | Age  Sex  Left ventricular ejection fraction  Hemoglobin  Platelet count  Estimated glomerular filtration rate  Race (non-hispanic white vs. Other)  Current dialysis, left main stenosis ≥50%  Proximal left anterior descending coronary artery stenosis ≥70%  Prior mi, endocarditis  Prior stroke or transient ischemic attack  Carotid stenosis  Prior peripheral arterial disease  Current or recent smoker  Diabetes  New york heart association class iv  Severe chronic lung disease  Use of home oxygen  Hostile chest  Porcelain aorta  Access site (femoral vs. Other)  Pacemaker  Previous implantable cardioverter defibrillator  Prior percutaneous coronary intervention  Prior coronary artery bypass grafting  Prior cardiac operators (≥2 vs. 1 vs. 0)  Prior aortic valve procedure  Prior non–aortic valve procedure, aortic etiology (degenerative vs. Other)  Valve morphology ([moderate/severe vs. Other]  Mitral insufficiency [moderate/severe vs. Other]  Tricuspid insufficiency moderate/severe vs. Other])  Acuity (elective vs. Urgent vs. Shock or inotropes or assist device vs. Emergency or salvage or cardiac arrest)  Discharge medications |
| --- | --- |
| Benedetto et al 2020 | Age  Female  Sex  New York Heart Association class  left ventricular ejection fraction  diabetes mellitus  smoking status  chronic obstructive  pulmonary disease  arterial hypertension (medically treated)  prior myocardial infarction  body mass index  creatinine  previous CVA  peripheral vascular disease  unstable angina  previous percutaneous coronary intervention  off-pump surgery  and total number of grafts |

**Table 2S.** (Continued)

| Taha et al 2020 | Sex  Age  Year of cardiac surgery  Body mass index  History of myocardial infarction  History of unstable angina  History of stable angina  Previous Percutaneous coronary intervention  History of diabetes mellitus  History of hypertension  History of chronic respiratory disease  History of peripheral arterial disease  History of cancer  History of chronic renal failure  History of renal replacement therapy  History of heart failure  Left ventricular ejection fraction  History of unspecified stroke  History of ischemic stroke  History of hemorrhagic stroke  History of transitory ischemic attack  History of liver disease  History of liver operation  History of pulmonary embolism  History of deep vein thrombosis  History of peripheral arterial embolism  History of intracranial bleeding  History of gastrointestinal bleeding  History of anemia  History of pericardial bleeding  History of pulmonary bleeding  CHA2DS2-VASc score  Treatment with diuretics  Treatment with Mineralocorticoid receptor antagonists  Treatment with Beta-blockers  Treatment with Angiotensin-converting enzyme inhibitors  Treatment with Angiotensin receptor blockers  Treatment with Calcium channel blockers  Treatment with Lipid lowering agents  Treatment with Antiplatelets  Treatment with Oral anticoagulants  Treatment with Corticosteroids  Treatment with Non-steroidal anti-inflammatory drugs  Treatment with Antiarrhythmic agents  Treatment with Insulin and Antidiabetic agents |
| --- | --- |
| Madsen et al 2021 | Age  Sex  Prior bleeding leading to hospitalization  *Except for ischemic stroke (excluded sex from the model) |

**Table 2S -** Covariates used in the adjusted cox regression models, by selected study.

**Table 3S.** Definitions of thromboembolic and bleeding events.

| Study | Thromboembolism | Major Bleeding |
| --- | --- | --- |
| Vora et al 2018 | **-** | Overt bleeding either associated with a drop in the haemoglobin level of at least 3.0 g/dL or requiring transfusion of two or three units of whole blood/RBC and does not meet criteria of life-threatening or disabling bleeding |
| Madsen et al 2021 | **-** | Requiring hospitalization |
| Benedetto et al 2020 | **-** | Haemorrhage requiring blood transfusion (type 3a), compromising patient hemodynamically (3b), requiring surgical reintervention (type 4), or resulting in patient death (type 5) |
| Taha et al 2020 | Composite of ischemic stroke, transient cerebral ischemia, and thrombosis or embolism in peripheral arteries | - |
| Butt et al 2018 |  | - |
| Butt et al 2019 |  | - |

**Table 3S.** Definition of thromboembolism and major bleeding events, by selected study.

**
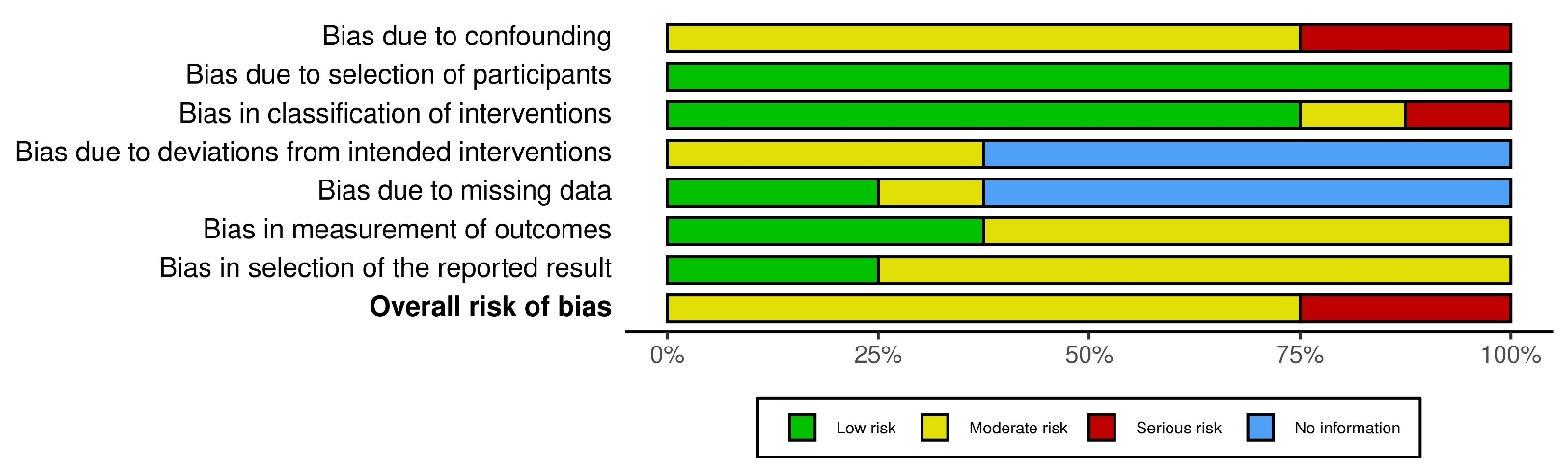
**

**Figure 1S**. 'Risk of bias' graph: review of the authors' judgements about each “Risk of bias” item presented as percentages across all included studies.


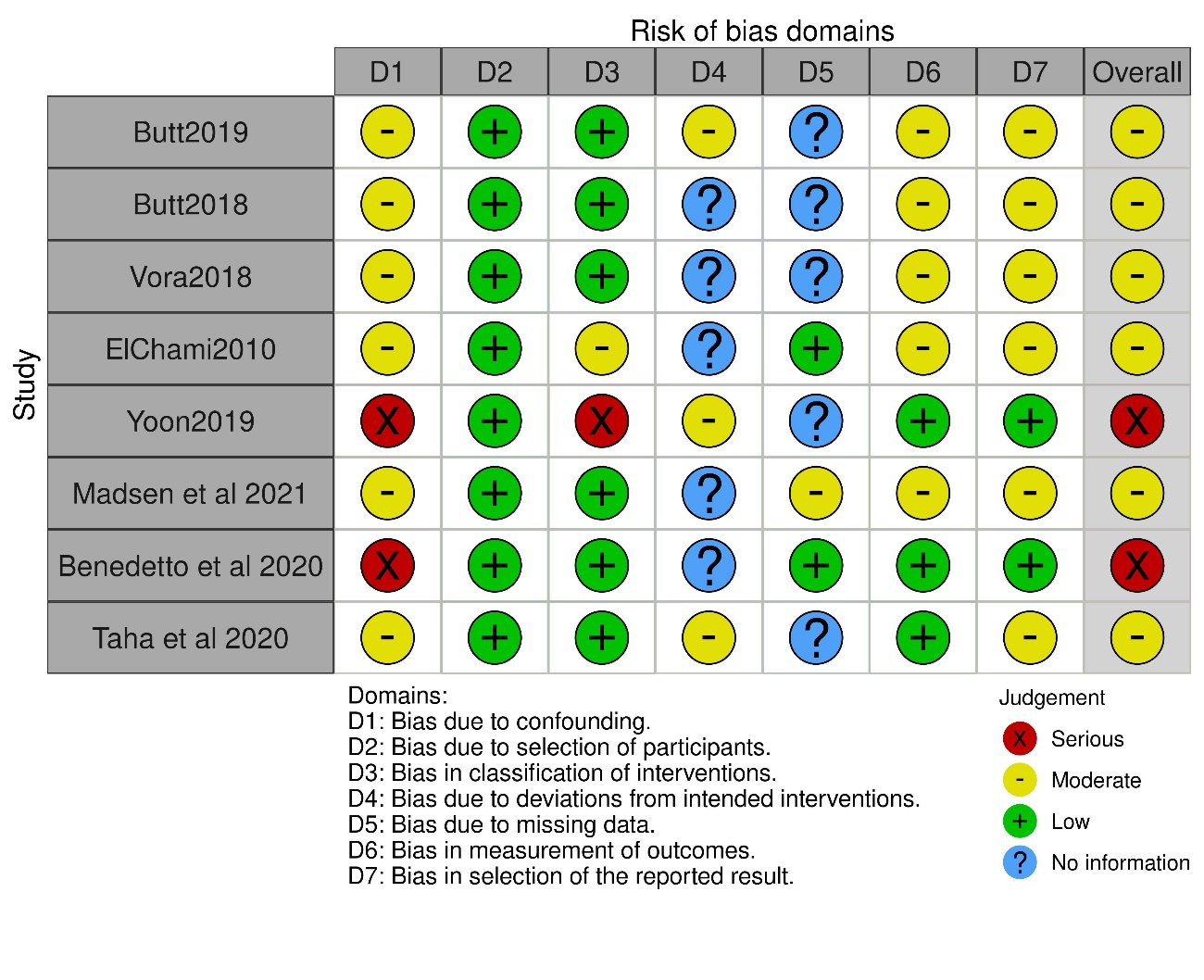


**Figure 2S**. “Risk of bias” summary: review of the authors' judgements about each “Risk of bias” item for each included study.
